# Supplementary material for: A Global Screen for Assembly State Changes of the Mitotic Proteome by SEC-SWATH-MS
Source: Cell Syst. 2020 Feb 26;10(2):133–155.e6. doi: 10.1016/j.cels.2020.01.001 (PMC7042714; doi:10.1016/j.cels.2020.01.001)

P42330 | AK1C3_HUMAN | AKR1C3 DDH1 HSD17B5 KIAA0119 PGFS
Monomer MW [kDa]: 36.853 Monomer expected elution fraction: 49

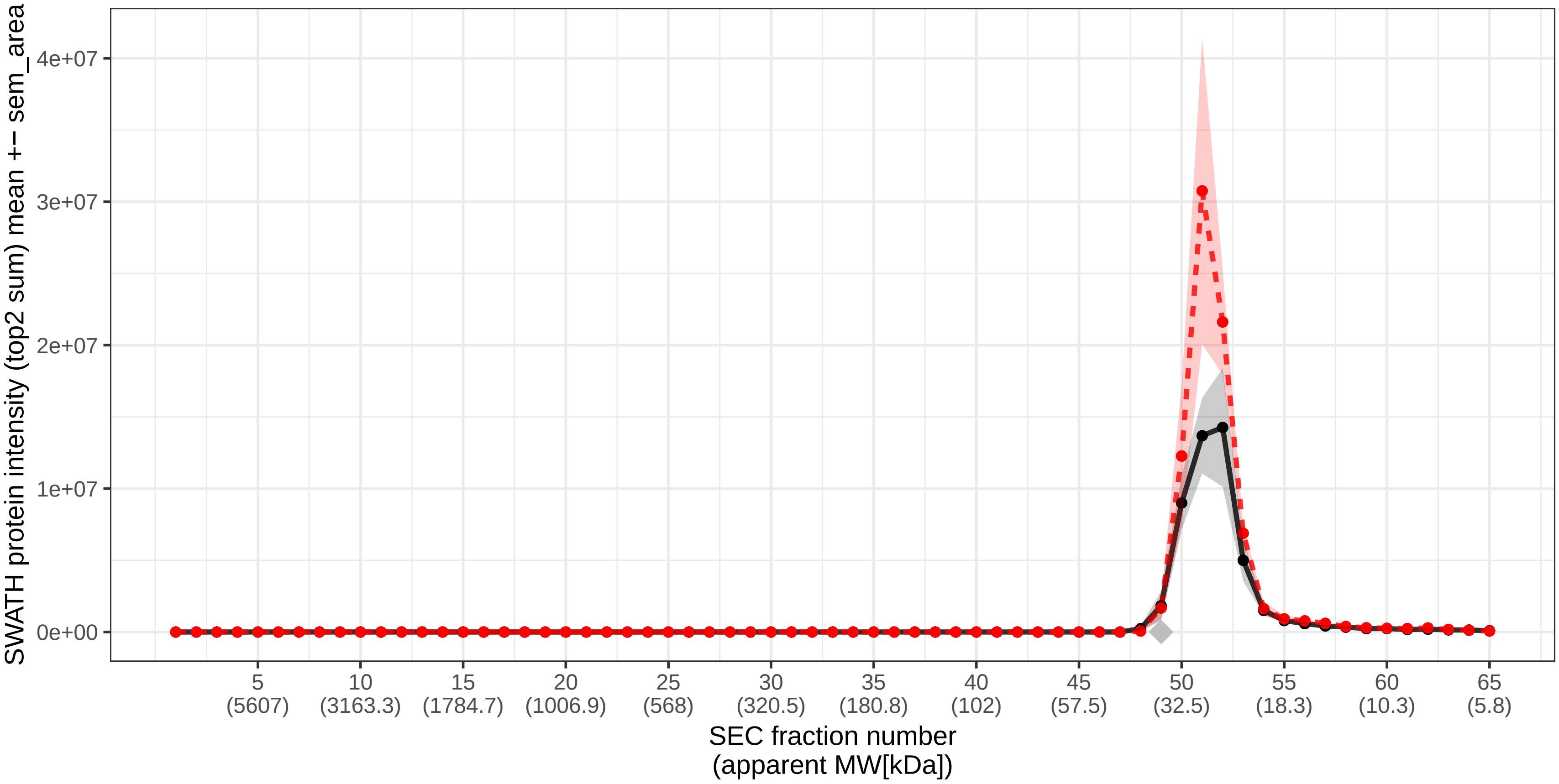

Supplement: Data S1. SEC-SWATH-MS Protein Chromatograms, Related to Figure 1 [file mmc6.zip › SECchrom_P42330_AK1C3_HUMAN_AKR1C3_DDH1_HSD17B5_KIAA0119_PG.pdf]
